# Thyroid Storm in a Patient With Alemtuzumab-Induced Graves’ Disease: A Case Report

**DOI:** 10.7759/cureus.24570

**Published:** 2022-04-28

**Authors:** Sara AlShehri, Sarah Alajmi, Aishah Ekhzaimy, Sadeem Aldawas, Maryam Alalwan

**Affiliations:** 1 Endocrinology, Diabetes and Metabolism, King Saud University, Riyadh, SAU; 2 Internal Medicine/Adult Endocrinology, King Saud University, Riyadh, SAU; 3 Endocrinology, Diabetes and Metabolism, King Saud University Medical City, Riyadh, SAU; 4 Internal Medicine, King Saud University Medical City, Riyadh, SAU; 5 Endocrinology and Diabetes, King Saud University Medical City, Riyadh, SAU

**Keywords:** graves’ disease, immune reconstitution therapy, thyrotoxic crisis, thyroid storm, alemtuzumab

## Abstract

Thyroid storm (TS) is a rare and life-threatening medical emergency, most commonly caused by Graves’ disease (GD). GD can be induced by immune reconstitution therapy (IRT) such as alemtuzumab (ALZ), a humanized monoclonal antibody against CD52, which is shown to be effective in the treatment of relapsing-remitting multiple sclerosis (RRMS). Here, we present a rare case of TS developing in a 39-year-old female with ALZ-induced GD, managed with antithyroid medication followed by thyroidectomy. There is some evidence that ALZ-induced GD may behave less aggressively than conventional GD. However, physicians should be aware that severe thyrotoxicosis and thyroid storm can happen, which requires prompt recognition and aggressive therapy.

## Introduction

Thyroid storm (TS) or thyroid crisis is a life-threatening emergency. Well-known triggers include undiagnosed hyperthyroidism, infection, surgery, acute illness, and rarely trauma [1]. Noncompliance with medication and factitious use of thyroxine (T4) have also been described in the literature as triggers for TS [2,3]. The mortality rate in hospitalized patients has declined over the past few years from 75% to 10%-30%; this is most likely due to improvement in early detection and intensive care measures. The leading cause of death is multiple organ failure, followed by congestive heart failure, respiratory failure, arrhythmia, disseminated intravascular coagulation, gastrointestinal perforation, hypoxic brain syndrome, and sepsis [1].

Multiple sclerosis (MS) is a chronic inflammatory condition characterized by inflammation, demyelination, and axonal degradation in the central nervous system (CNS), affecting approximately 2.5 million individuals globally. MS therapy has undergone a paradigm shift in recent years due to emerging medications such as alemtuzumab (ALZ), a humanized monoclonal antibody targeting CD52, which is a surface molecule that is mostly expressed on B and T lymphocyte cells and has a largely unclear function [4]. It has been licensed for the treatment of relapsing-remitting multiple sclerosis (RRMS) in over 30 countries since it was approved by the United States Food and Drug Administration (FDA) in November 2014 [4,5]. Alemtuzumab proved superiority over interferon β-1a (IFNβ-1a), a disease-modifying treatment (DMT) for RRMS that has been in use for several years [6,7]. The strong effectiveness of alemtuzumab, which has been shown to have considerable benefits on clinical and radiological disease outcome measures, is counterbalanced by a high frequency of serious adverse events.

Autoimmune thyroid events (ATEs) are the most frequent adverse events after ALZ therapy. Across the several types of ATEs, Graves’ disease (GD) was the most prevalent thyroid dysfunction, accounting for more than half of the cases [4]. Around 15% of patients with ALZ-induced GD have a fluctuating and unpredictable course [8]. This is attributed to the proportion of thyroid-stimulating hormone (TSH) receptor-stimulating antibody (TSAb) and TSH receptor-blocking antibody (TBAb), which play a major role in the “pendulum swinging” of thyroid function [8]. Therefore, ALZ-induced GD cannot be deemed to have a more favorable prognosis than conventional GD. However, by implementing a safety monitoring program, these autoimmune occurrences may be detected and managed early. Here, we describe a case of a 39-year-old female diagnosed with ALZ-induced GD who presented with a thyroid storm in the setting of urosepsis and medication noncompliance. The management was further confounded by high liver enzymes caused by severe thyrotoxicosis and antithyroid therapy, as well as a large goiter that required thyroidectomy. This case highlights the value of early detection and treatment and the importance of patient education regarding the possible side effects of ALZ.

## Case presentation

**Figure 1:**
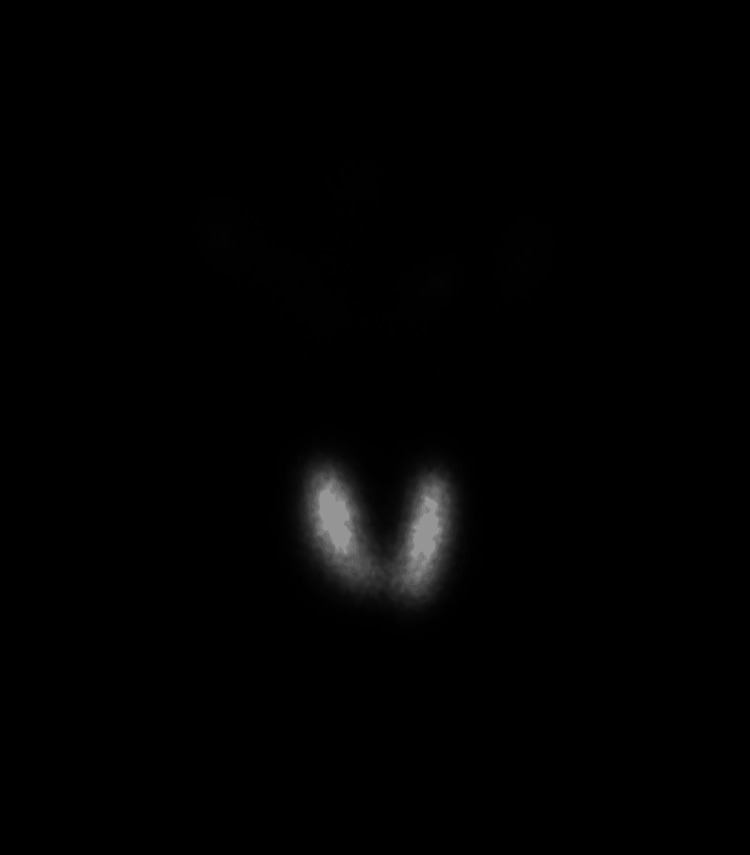
Thyroid scan showing diffuse enlargement with markedly increased uptake of both thyroid glands.

This case is of a 39-year-old female, a nonsmoker, known to have RRMS complicated by physical dependency and neurogenic bladder with recurrent urinary tract infections (UTIs). The patient was initially managed with natalizumab for her RRMS, which was stopped due to her infection with John Cunningham virus (JCV), and was then started on ALZ. Past medical history includes depression/anxiety disorder managed with quetiapine. She presented to the emergency room 13 months after the first dose of ALZ with palpitation, excessive sweating, diarrhea, and oligomenorrhea. She was found to have a suppressed thyroid-stimulating hormone (TSH) (0.007 mIU/L; normal values: 0.250-5 mIU/L) and elevated T4 (51.6 pmol/L; normal values: 11.5-22.7 pmol/L) with positive thyroid peroxidase antibody (TPO) 183 units (>100 positive); TSH receptor-stimulating antibody (TSAb) was not available in our facility. A thyroid scan demonstrated a diffusely enlarged thyroid gland with increased tracer uptake compatible with GD (Figure 1). Our impression was ALZ- induced GD (without thyroid storm). She was started on carbimazole 20 mg three times a day (TID) and propranolol 40 mg TID. After initiating carbimazole, her liver enzymes increased twofold, and the patient was not compliant due to underlying psychiatric problems and lack of family support. Therefore, we decided to proceed with radioiodine ablation therapy after the stabilization of her thyroid function. She was discharged home on carbimazole and propranolol. In the subsequent follow-up visits, her thyroid function improved, and she became euthyroid but was still having recurrent UTIs. After the UTIs subsided, her primary neurologist decided to give her a second cycle of ALZ as she was euthyroid on carbimazole.

One month later, the patient presented to our emergency room with decreased appetite, nausea, and vomiting. Her sister reported that she was not taking her antithyroid drugs regularly for two weeks prior to her presentation. On examination, she was febrile at 38.2°C, tachycardic reaching 140 beats/minute (regular), with a blood pressure of 138/87 mmHg and saturating 98% on room air. The patient was agitated, anxious, alert, and oriented. She was diaphoretic, with mild peripheral edema and pulmonary crackles. Her thyroid was diffusely enlarged with no signs of Graves’ ophthalmopathy or pretibial myxedema. She scored 65 on the Burch-Wartofsky Point Scale for thyroid storm and was admitted to the intensive care unit (ICU), with both the endocrine and neurology teams involved in her care.

Laboratory investigation revealed TSH of 0.009 mIU/L, triiodothyronine (T3) of 26.5 pmol/L (normal values: 3.39-5.82 pmol/L), and T4 of 65 pmol/L (Table 1). Blood and urine cultures were collected. An initial electrocardiogram (EKG) revealed sinus tachycardia and nonspecific ST-T wave changes. She was started on carbimazole 20 mg every six hours, propranolol 40 mg every eight hours, hydrocortisone 100 mg every eight hours, potassium iodide, and empiric antibiotics with intravenous fluids. The blood culture result was negative, but the urine culture revealed extended-spectrum beta-lactamases *Escherichia coli* with an upgrade in her antibiotics to meropenem. Shortly after, the patient developed respiratory distress with a drop in her oxygen saturation to 20% on room air. The monitor showed asystole, and there was no pulse, so cardiopulmonary resuscitation was started. The patient was revived after three cycles and then was intubated and sedated.

**Table 1:** Patient’s laboratory values. TSH: thyroid-stimulating hormone; TPO: thyroid peroxidase antibody

Laboratory test	Reference range	Patient’s value on initial admission	Patient’s value on the next admission
TSH	0.250-5 mIU/L	0.007 mIU/L	0.009 mIU/L
T4	11.5-22.7 pmol/L	51.6 pmol/L	65 pmol/L
T3	3.39-5.82 pmol/L	-	26.5 pmol/L
TPO	>100 units positive	183	-

**Figure 2:**
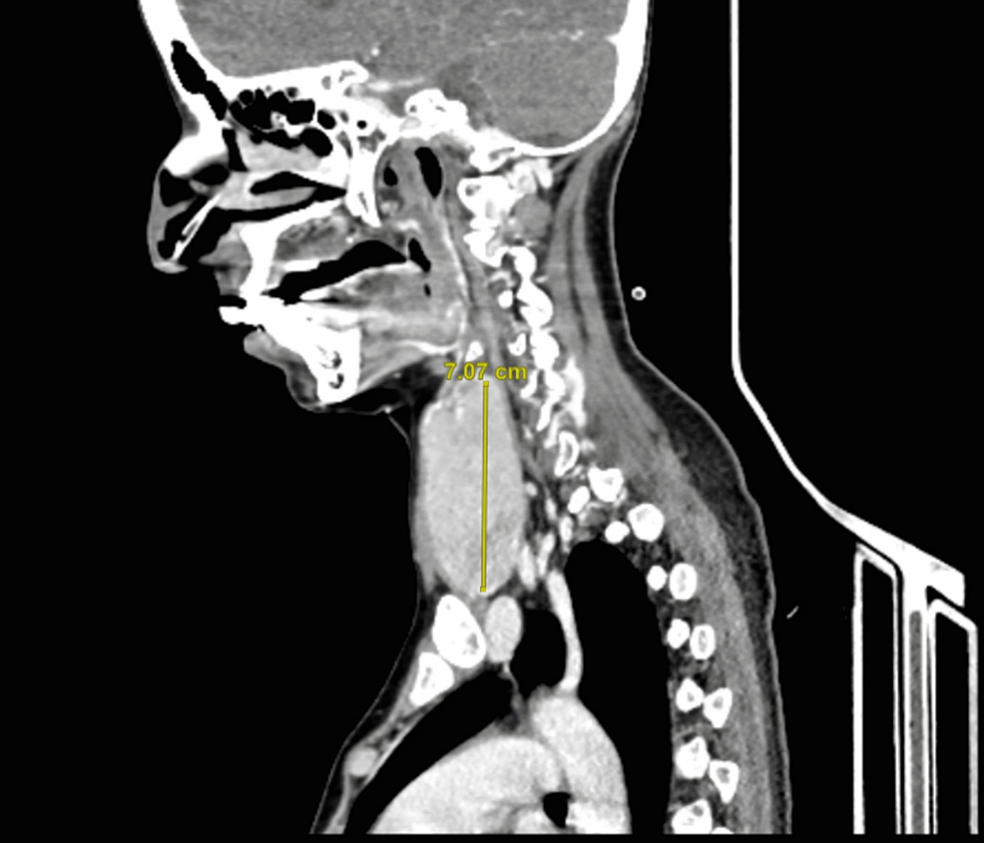
Neck CT (sagittal view) showing markedly enlarged thyroid gland with no retrosternal extension. The yellow line delineates the size of the thyroid gland. CT: computed tomography

**Figure 3:**
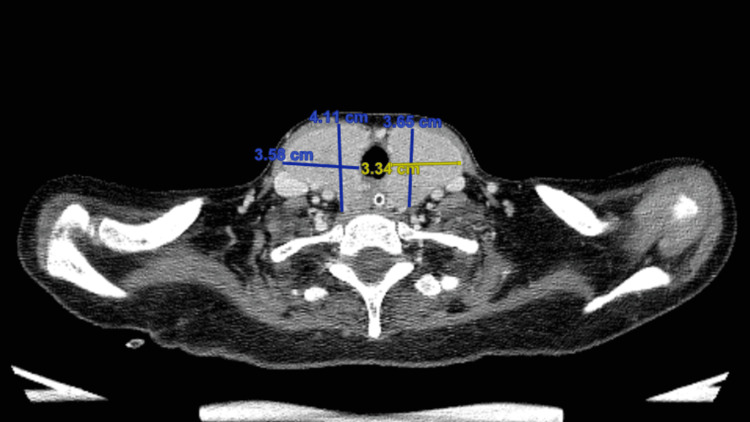
Neck CT (coronal view). The yellow and blue lines delineate the size of the thyroid gland. CT: computed tomography

On day 5, the ICU team tried to extubate her, but unfortunately, two hours after extubation, she was reintubated as she developed respiratory distress. Given that it was difficult to wean her from the ventilator, a tracheostomy was planned, but it was not possible to proceed due to the large goiter. Neck computed tomography (CT) was done to rule out retrosternal extension (Figure 2), which showed kissing retro-esophageal thyroid causing compression on the esophagus (Figure 3). Due to this goiter, thyroidectomy was planned after the stabilization of her thyroid function. On day 18, T4 normalized; however, the patient was hypotensive and required inotropic support for hospital-acquired pneumonia.

**Figure 4:**
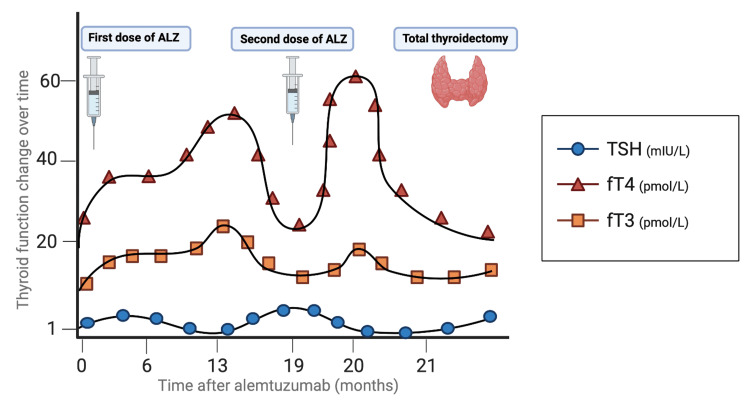
Thyroid function tests throughout the patient’s course revealed a substantial increase in free T3 and free T4 and a decline in TSH 13 months after the first dose and one month after the second dose of alemtuzumab. TSH: thyroid-stimulating hormone Note: 0 represents the first day of alemtuzumab treatment. *Created with BioRender.com

Finally, on day 29, she underwent a total thyroidectomy with tracheostomy insertion. Steroids and β-blockers were gradually tapered. She was then maintained on levothyroxine 100 mcg daily (Figure 4). The surgery was complicated by a left recurrent laryngeal nerve injury. The patient was then transferred to a long-term facility for continuity of care.

## Discussion

Alemtuzumab is a humanized monoclonal antibody targeting CD52, a surface molecule that is mainly expressed on B and T cells. It acts by inducing rapid and prolonged depletion of both B and T lymphocytes, causing pan-lymphopenia with consequent immunosuppression, followed by a phase of immune reconstruction [9]. ALZ has demonstrated great effectiveness in clinical phase II and III trials greater than that of interferon β for patients with RRMS [6,7]. However, ALZ is linked to a number of serious adverse effects, most notably autoimmune adverse events, which mostly impact thyroid function. It is estimated that around 30%-40% of individuals who were treated with ALZ developed thyroid dysfunction; this was attributed to the immune reconstitution phase with the restoration of the lymphocyte network over the next 2-5 years that can trigger the production of autoantibodies against the thyroid gland [8].

Thyroid dysfunction following immune reconstitution therapy can range from mild, self-limited thyroiditis to rarely severe thyrotoxicosis. Graves’ disease associated with ALZ treatment may behave less aggressively than conventional GD. The CAMMS223 trial revealed that 22% of ALZ-treated patients developed GD and 78% of those who developed GD recovered, either spontaneously or after antithyroid therapy [7]. Overall, the authors concluded that there were only a few serious episodes of thyroid dysfunction. The same was observed in the CARE-MS II trial, where all cases of GD were managed with antithyroid drugs, and only two patients required radioiodine therapy or surgery [6]. Smoking, family history of thyroid disease, and high titer of thyroid antibodies are linked to an increased risk of having severe disease [10]. Our patient was not a smoker, and she has no family history of thyroid disease; however, she had a high TPO titer. She was initially managed with carbimazole and planned for radioiodine ablation given her history of noncompliance, lack of family support, psychiatric illness, and derangement of her liver function; unfortunately, this was not pursued due to her frequent admissions for urosepsis.

Thyroid storm is a rare and life-threatening endocrine emergency that requires prompt recognition and treatment. Infection and medication noncompliance are the most common triggers for thyroid storm. It accounts for 1%-2% of hospital admissions [1]. The diagnosis of TS is challenging as there is no indicative cutoff point for thyroxine (T4), triiodothyronine (T3), or thyroid-stimulating hormone (TSH) to diagnose patients with TS. Thus, it is mostly dependent on clinical scoring systems, which can aid in the diagnosis. To date, there are no standardized clinical tools for diagnosis. The Burch-Wartofsky Point Scale [11] is based on clinical criteria that include the precipitating factors and the severities of symptoms of multiple organ decompensation (thermoregulatory dysfunction, tachycardia/atrial fibrillation, disturbances of consciousness, congestive heart failure, and gastro-hepatic dysfunction); a score of 45 or higher is strongly indicative of thyroid storm, as in our patient who had a score of 65 (points for a temperature of 38.3°C, a pulse of 140 beats/minute, agitation, bibasilar rales, and precipitant history). On the other hand, the Japanese Thyroid Association (JTA) diagnostic criteria for thyroid storm requires the presence of thyrotoxicosis with a combination of other symptoms (CNS manifestation, fever, tachycardia, heart failure, and gastro-hepatic manifestation) [12]. Applying these criteria to our patient revealed a diagnosis of definitive thyroid storm (high free T3 and free T4 with agitation and tachycardia).

Due to the high mortality rate associated with TS, therapy should begin as soon as the diagnosis is suspected. These critically sick patients are typically triaged to an intensive care unit to allow for careful patient monitoring and the deployment of aggressive treatment options in a timely manner. A multidisciplinary team approach is critical to properly offer all therapy options to the patient. Treatment options should not only aim to decrease the synthesis and release of the thyroid hormone but also to mitigate the effect of circulating thyroid hormone and avoid end-organ damage [13]. Antithyroid medications such as thionamide and propylthiouracil (PTU) are considered the first-line therapeutic option in individuals with ALZ-induced GD [14]. These medications suppress thyroid peroxidase, a paramount enzyme involved in the conversion of thyroglobulin to T3 and T4, and reduce follicular cell proliferation. Propylthiouracil is preferred during thyroid storm due to its additional effect of lowering peripheral conversion of T4 to T3 [13]. In our patient, PTU was not used as she had deranged liver enzymes.

Iodine administration has been demonstrated to limit the proteolytic release of iodothyronines (T3 and T4) from thyroglobulin, hence decreasing the release of a preformed hormone, in addition to its effect on hormone synthesis [1]. It is crucial to take iodine at least 60 minutes after thionamides are administered to avoid iodine functioning as a substrate for additional thyroid hormone synthesis, which could exacerbate the hyperthyroid state. Thionamides must also be maintained throughout iodine therapy to minimize iodine organification and prevent thyroid hormone production. When administered appropriately, the combined treatment with thionamides and iodine can reduce serum T4 levels closer to the normal range within 4-5 days [1].

Propranolol, a nonselective β-adrenergic receptor antagonist, is the most commonly used β-blocker in thyroid storm, as it inhibits the peripheral conversion of T4 to T3 [13]. Glucocorticoid is frequently used to reduce peripheral conversion of T4 to T3 in addition to preventing adrenal insufficiency. Treating TS requires addressing the underlying triggering cause. Infection (urosepsis) was one of the precipitating factors in our patient, which was managed with antibiotics. Noncompliance to antithyroid medication is another factor that was difficult to control given her history of psychiatric illness.

Surgery is considered the last resort in TS [1]. Radioiodine ablation was initially considered for our patient as she had elevated liver enzymes, as well as the concern about her compliance to antithyroid medication as she had an underlying psychiatric illness and lack of family support; however, due to her frequent admission with recurrent UTIs, it was postponed. After then, she was unstable to go for radioiodine ablation, and the ICU team struggled to wean her from the ventilator, so she required tracheostomy insertion, which was impossible in light of the presence of a large goiter, which we believed is the cause of her respiratory distress as her sister mentioned that for the last month she was complaining from dysphagia and she had choking multiple times, for that decision shifted toward thyroidectomy, which carries a high risk, especially in the case of uncontrolled thyrotoxicosis. An experienced surgeon with expert facility for the medical management of patients pre- and postoperatively in the ICU setting is required. Scholz et al. examined the literature on the surgical outcome of early thyroidectomy for patients with TS (not severe thyrotoxicosis), and they include their own series of 10 patients [15]. The long-term overall mortality of patients treated with thyroidectomy was 10% (five of 49) with a wide range from 0% to 20% in different series. However, this data was retrospective, which could be affected by selection bias; also, they only included old-age patients.

Based on the European Thyroid Association guidelines on the management of thyroid dysfunction following immune reconstitution therapy published in 2019 [14], thyroid immune-related adverse reactions have a late and variable onset, usually occurring 6-23 months after the last dose of ALZ. Patients should have a baseline thyroid function test before initiating ALZ and then be monitored frequently every three months or sooner if the patient develops symptoms of thyroid dysfunction. Thyroid function monitoring should continue even after stopping ALZ for a total of four years from the last ALZ dose. After this period, testing should be performed based on symptoms and signs suggestive of thyroid dysfunction.

## Conclusions

In conclusion, with the new era of target-specific immunotherapies, frequent monitoring for thyroid dysfunction and early intervention enable effective risk management. It is thought that ALZ-induced GD behaves less aggressively than conventional GD, as spontaneous or drug-induced remission in ALZ- induced GD may be more likely. However, physicians should be aware that severe thyrotoxicosis and thyroid storm can occur, which requires prompt recognition and aggressive therapy. It is crucial to educate the patient about the possible side effects of these medications, raise their awareness of the symptoms of thyroid disorders, and acknowledge their psychiatric disorder and family support as important factors for medication compliance.
